# Supplementary material for: Medium levels of transcription and replication related chromosomal instability are associated with poor clinical outcome
Source: Sci Rep. 2021 Dec 6;11:23429. doi: 10.1038/s41598-021-02787-x (PMC8648741; doi:10.1038/s41598-021-02787-x)
Supplement: Supplementary file 1 — Supplementary Figures. [file 41598_2021_2787_MOESM1_ESM.pptx]

## Slide 1
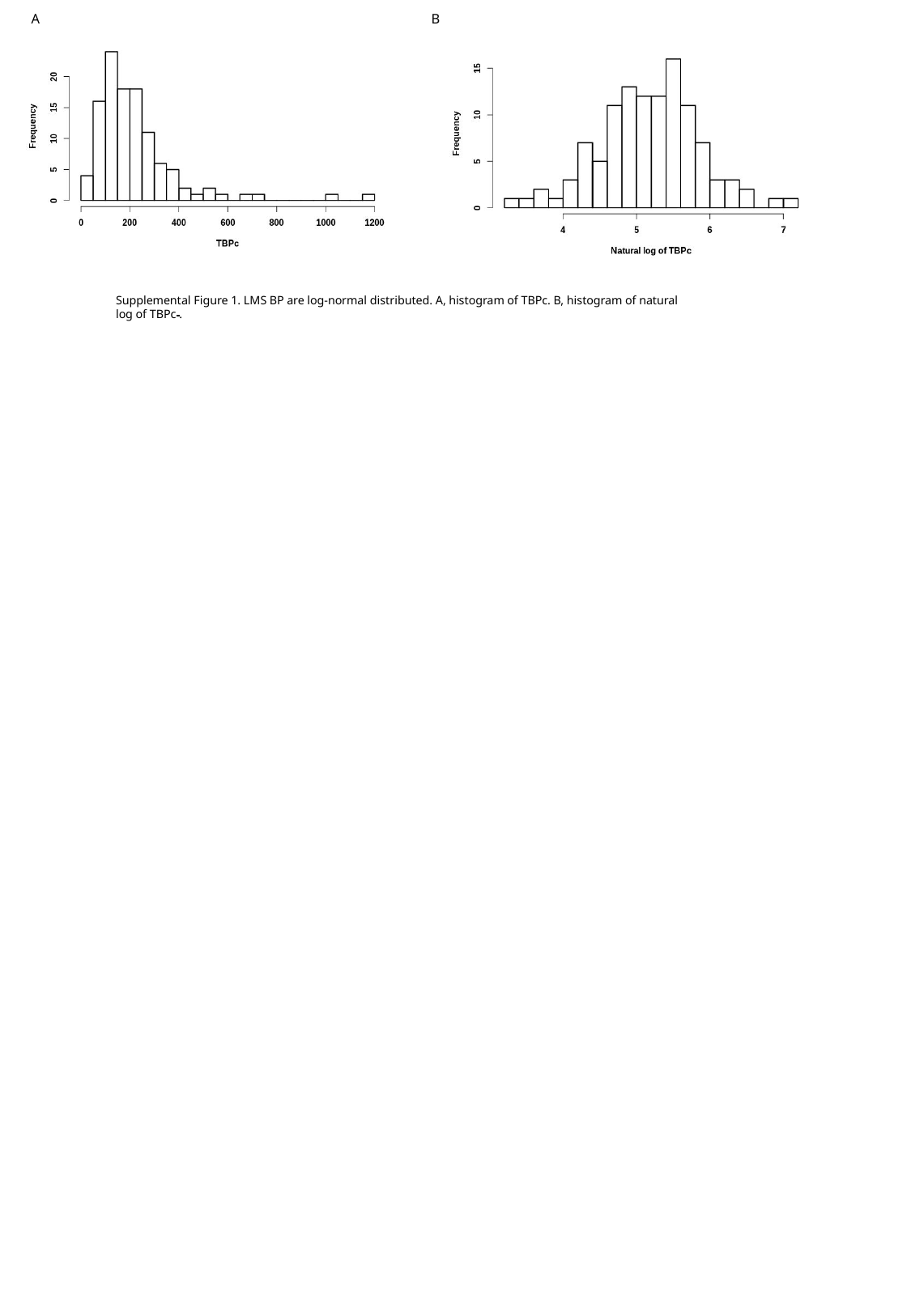

A
B
Supplemental Figure 1. LMS BP are log-normal distributed. A, histogram of TBPc. B, histogram of natural log of TBPc .

## Slide 2
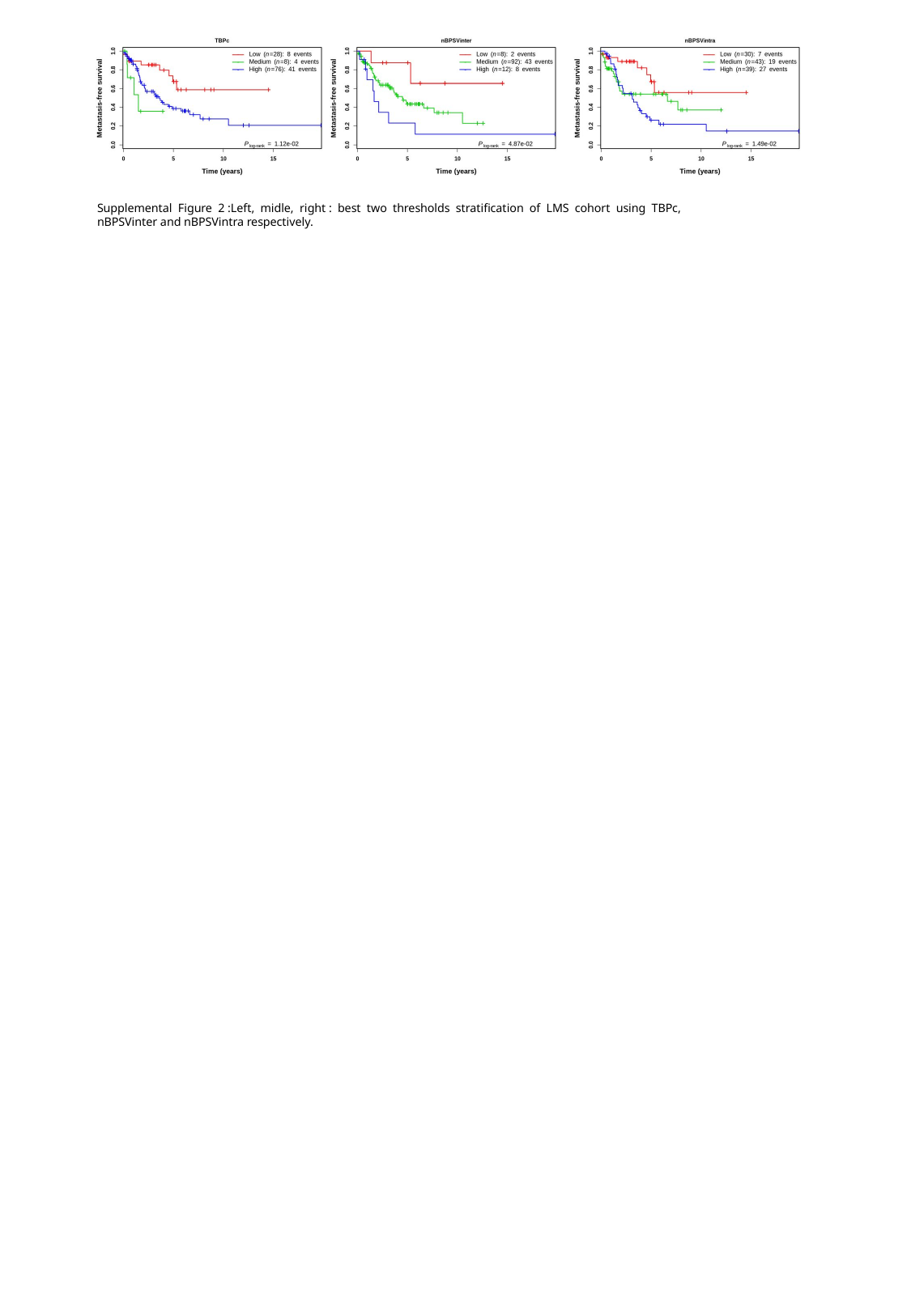

Supplemental Figure 2 :Left, midle, right : best two thresholds stratification of LMS cohort using TBPc, nBPSVinter and nBPSVintra respectively.

## Slide 3
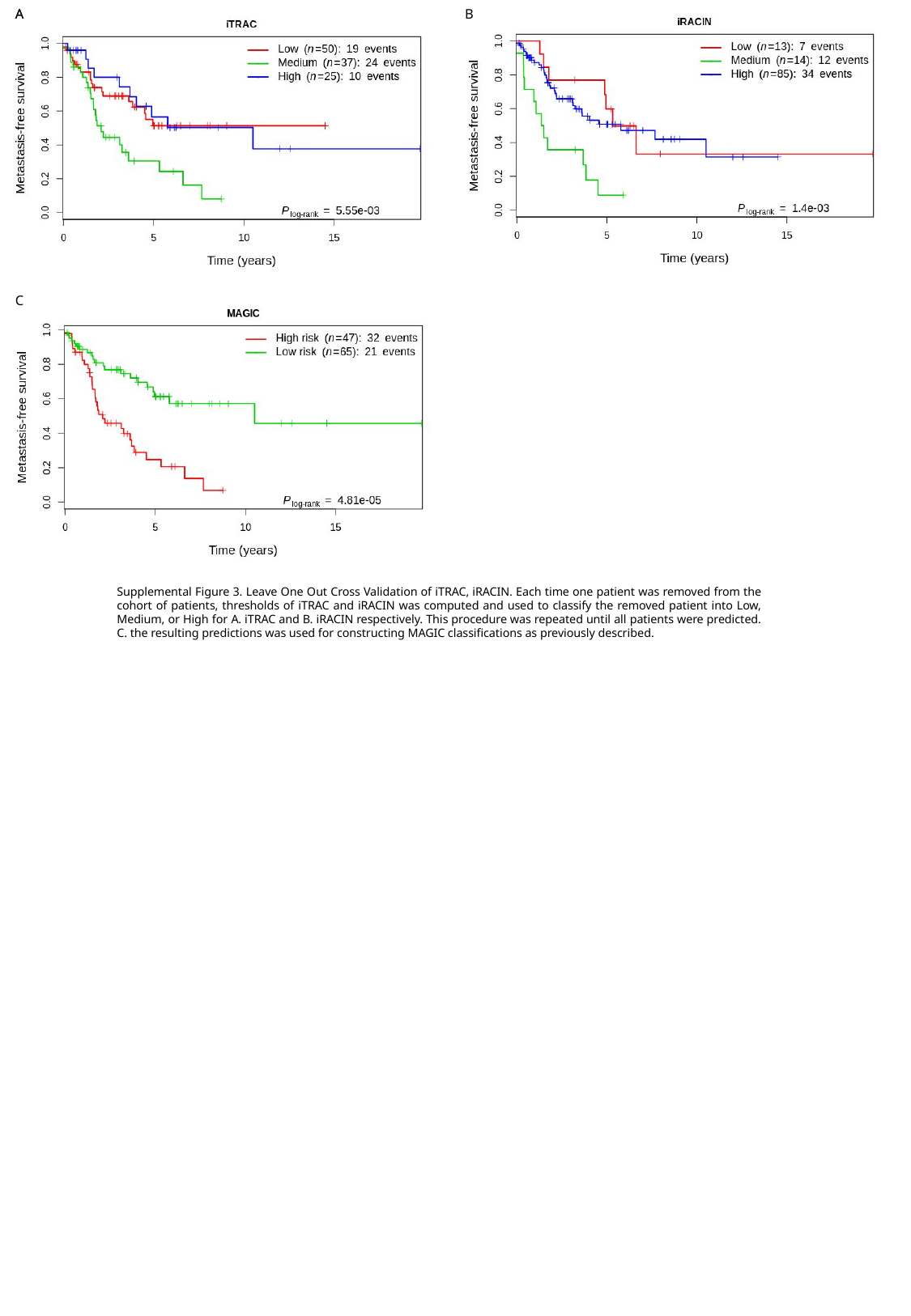

A
A
B
C
Supplemental Figure 3. Leave One Out Cross Validation of iTRAC, iRACIN. Each time one patient was removed from the cohort of patients, thresholds of iTRAC and iRACIN was computed and used to classify the removed patient into Low, Medium, or High for A. iTRAC and B. iRACIN respectively. This procedure was repeated until all patients were predicted. C. the resulting predictions was used for constructing MAGIC classifications as previously described.

## Slide 4
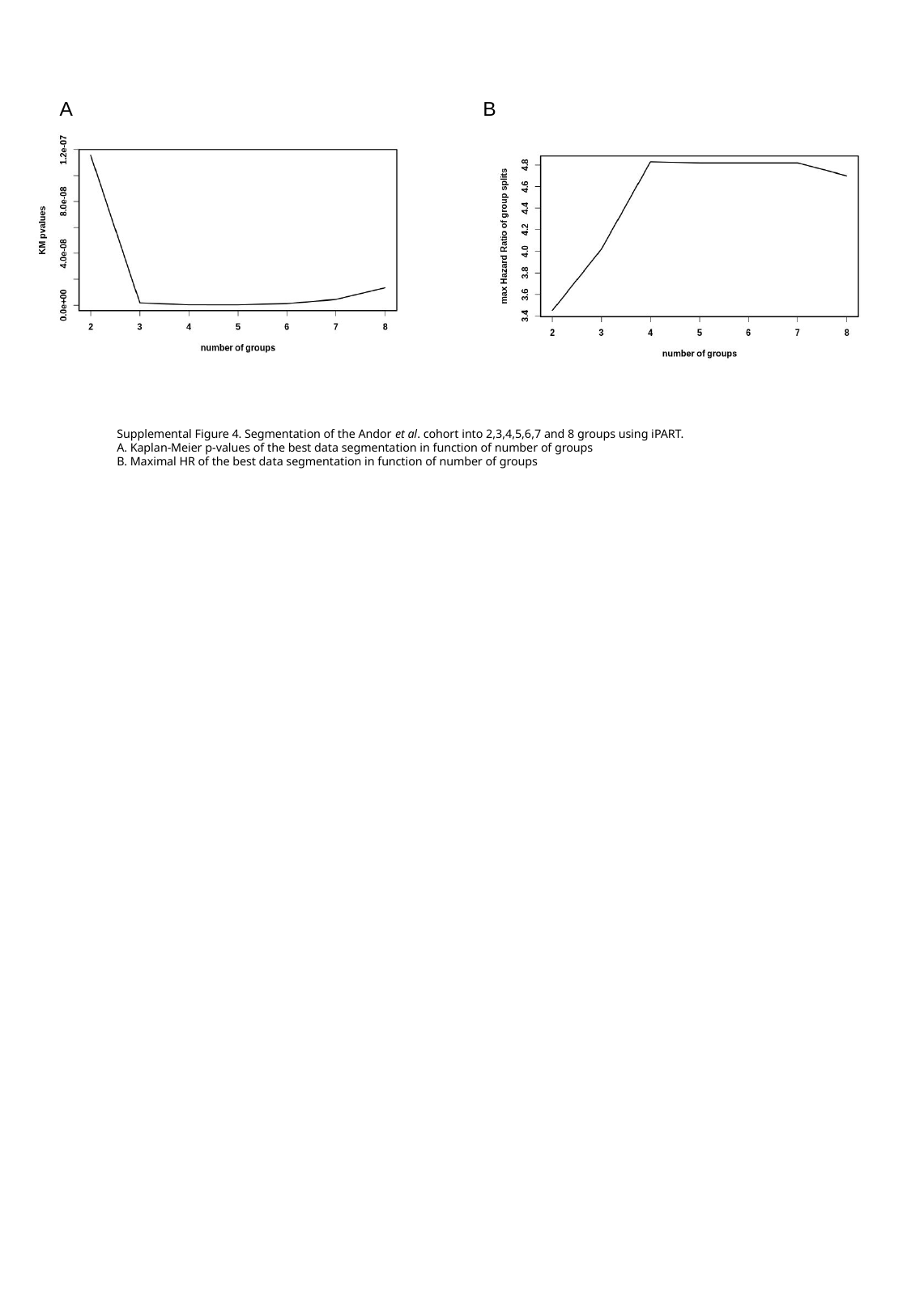

A
B
Supplemental Figure 4. Segmentation of the Andor et al. cohort into 2,3,4,5,6,7 and 8 groups using iPART.
A. Kaplan-Meier p-values of the best data segmentation in function of number of groups
B. Maximal HR of the best data segmentation in function of number of groups

## Slide 5
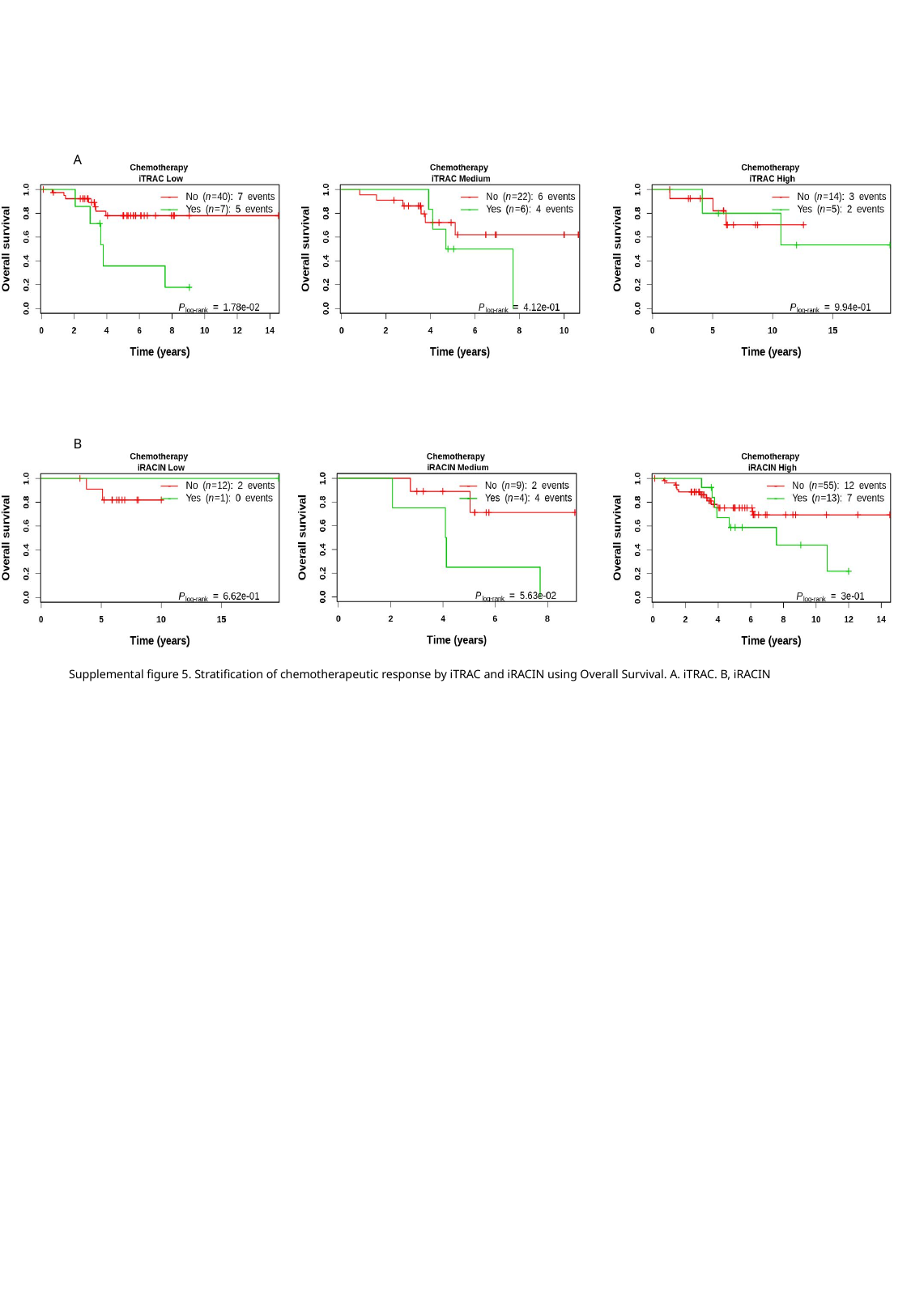

A
B
Supplemental figure 5. Stratification of chemotherapeutic response by iTRAC and iRACIN using Overall Survival. A. iTRAC. B, iRACIN
